# Supplementary material for: Embryo biosensing by uterine natural killer cells determines endometrial fate decisions at implantation
Source: FASEB J. 2021 Mar 22;35(4):e21336. doi: 10.1096/fj.202002217R (PMC8251835; doi:10.1096/fj.202002217R)
Supplement: Supplementary file 1 — Supplemental figures and tables [file FSB2-35-0-s001.docx]

**Supplementary Information**

**Embryo biosensing by uterine natural killer cells determines endometrial fate decisions at implantation**

Chow-Seng Kong, Alexandra Almansa Ordoñez, Sarah Turner, Tina Tremaine, Joanne Muter, Emma S. Lucas, Emma Salisbury, Rita Vassena, Gustavo Tiscornia, Ali A. Fouladi-Nashta, Geraldine Hartshorne, Jan J. Brosens and Paul J. Brighton.

**Figure S1**. Expression of genes coding HA synthase and catabolic enzyme isoforms in undifferentiated EnSC (day 0) and cells decidualized with 8-br-cAMP and MPA (C+M) for 4 days. Individual data points from 3 biological repeat experiments are shown with bar graphs denoting mean ± SEM.

**Figure S2.** BCM does not impact on decidual subpopulations. Primary EnSC were first decidualized with C+M for 6 days and then exposed to UCM, BCM^+^ and BCM^-^, diluted 1 in 7.5 in differentiation medium (i.e. containing C+M). After 48 h, the cultures were harvested. SA-β-Gal activity and clusterin secretion mark the level of senescent decidual cells whereas sST2 secretion is confined to decidual cells. Individual data points from 3 biological repeat experiments are shown with bar graphs denoting mean ± SEM. Different letters indicate statistical differences (*P* < 0.05) between groups (one-way ANOVA and Dunnett’s multiple comparison test).

**Table S1. Patient demographics.**

| Endometrial biopsies used for EnSC cultures | | | | |
| --- | --- | --- | --- | --- |
| Figure | **(n)** | **Age** | **BMI** | **LH+** |
| 1A-B | 4 | 31.5 (28.8-36) | 27 (24.9-29.3) | 8 (8-8.3) |
| 1C | 5 | 31 (31-32.5) | 23 (22.5-24.5) | 9 (9-10) |
| 1E-G | 3 | 38 (35.5-38) | 23 (21.5-24) | 9 (8-9.5) |
| 2G | 3 | 33 (31.5-36.5) | 23 (22-24) | 9 (8-9) |
| 2H-I | 3 | 34 (33-35.5) | 29 (29-29.8) | 7 (7-8.5) |
| 3A | 7 | 33.5 (30.8-39.8) | 24 (23-24) | 7 (7-10) |
| 3B | 5 | 35.5 (35-37.8) | 22 (21.3-22.8) | 8 (7-9) |
| 3C | 4 | 35.5 (32.8-38) | 24 (22.5-26) | 8.5 (6.75-10.5) |
| 4B | 9 | 36 (34-40) | 25 (22.5-31) | 9 (8-10) |
| 4C | 4 | 35 (33-37.5) | 26 (24.5-30.6) | 9 (8-10) |
| S2 | 3 | 36 (34-38.5) | 23.3 (22.2-25.4) | 10 (9-10.5) |
| Endometrial biopsies used for uNK cell isolation | | | | |
| 2B-C | 12 | 36 (32-37) | 23 (21.8-23.8) | 9 (8.5-9.3) |
| 2D | 3 | 43 (39-43) | 21.8 (20.9-21.9) | 7 (6.5-8.5) |
| 2E | 4 | 33 (29.5-35.8) | 25 (23.5-27) | 10 (9.3-10.3) |
| 2G | 4 | 37.5 (36.8-38.8) | 25.95 (21.8-29) | 6.8 (6.8-8.5) |
| 2I | 4 | 35.5 (34.3-37.3) | 23 (22-24) | 9 (9-9.5) |
| 3A | 9 | 36 (32-40) | 27 (24-29) | 9 (8-9) |
| 3B | 6 | 34 (31.3-39) | 27.4 (24.8-30.5) | 8.5 (7.3-9) |
| 3C | 12 | 33 (31-37.8) | 29 (24-28.8) | 9 (7-10) |
| 4B | 11 | 35 (33-38) | 24 (22.5-25.5) | 7 (6-7) |
| 4C | 11 | 32 (31.5-33.5) | 23 (22.3-23.5) | 6 (6-6.5) |

All data are median (interquartile range, Q1-Q3).

**Table S2. Embryo grading and patient demographics** **(Fig. 4E-F).**

|  | Good Quality (n=23) | Poor Quality (n=20) | *P* value |
| --- | --- | --- | --- |
| Grade | ≥BB | <BB |  |
| Age* | 33 (31-35) | 36 (31.5-39.3) | 0.314 |
| IVF | 17 | 6 |  |
| ICSI | 6 | 14 |  |

|  | Pregnant (n=20)^ | Non-Pregnant (n=22) | *P* value |
| --- | --- | --- | --- |
| Age* | 32.1 (30-34) | 36.3 (33-40) | 0.003 |
| IVF | 16 | 6 |  |
| ICSI | 4 | 16 |  |

*median (interquartile range, Q1-Q3)

^ One ectopic pregnancy excluded

Significance determined by *t*-test
